# Neuroplasticity and Clinical Practice: Building Brain Power for Health

**DOI:** 10.3389/fpsyg.2016.01118

**Published:** 2016-07-26

**Authors:** Joyce Shaffer

**Affiliations:** Department of Psychiatry and Behavioral Sciences, University of WashingtonSeattle, WA, USA

**Keywords:** neuroplasticity, brain plasticity, neurogenesis, health psychology, healthy aging, cognitive, computerized cognitive training, cognition

## Abstract

The focus of this review is on driving neuroplasticity in a positive direction using evidence-based interventions that also have the potential to improve general health. One goal is to provide an overview of the many ways new neuroscience can inform treatment protocols to empower and motivate clients to make the lifestyle choices that could help build brain power and could increase adherence to healthy lifestyle changes that have also been associated with simultaneously enhancing vigorous longevity, health, happiness, and wellness. Another goal is to explore the use of a focus in clinical practice on helping clients appreciate this new evidence and use evolving neuroscience in establishing individualized goals, designing strategies for achieving them and increasing treatment compliance. The timing is urgent for such interventions with goals of enhancing brain health across the lifespan and improving statistics on dementia worldwide.

## Introduction

Since 2011, our Director of NIMH has encouraged “the 21st century discipline of clinical neuroscience” to include brain-plasticity based strategies in providing clinical care (White, 2011). Neuroplasticity, the capacity of brain cells to change in response to intrinsic and extrinsic factors, can have negative or positive influence at any age across the entire lifespan. How can these factors be influenced in clinical settings? In keeping with the increasing shift in focus from illness to what maximizes wellness, psychologists are uniquely trained to use evidence based behavioral techniques as effective methods for driving neuroplasticity in a positive direction.

Search efforts for this review were primarily focused within the PubMed database using combinations of keywords listed above as well as names of prominent researchers in the field. Also, bibliographies of these articles and related reviews were searched. Studies accepted for review included animal as well as human studies. Studies reviewed met rigorous scientific standards, were almost exclusively broad review articles or random controlled trials, and were included without a specified time span. Periodic searches of this nature were conducted between 2005 and 2016.

It has been estimated that dementia has been detected at the rate of one new person about every 7 s around the world (Ferri et al., 2005). Brookmeyer et al. (2007) estimate that success in delaying the onset of dementia by as little as a year could reduce the global burden of Alzheimer’s disease by as much as 9,200,000 cases in 2050, a number that makes driving brain plasticity in a positive direction a top priority worldwide.

The focus of this article is to review the scientific foundation and recent progress in research of neuroplasticity; relate that to the various ways these findings can influence treatment; propose ways treatment protocols could increase adherence to brain plasticity based therapeutics; and suggest research questions going forward. Before proceeding on how to impact the so called “normal age-related cognitive decline,” it is important to consider recent research on the myth of cognitive decline (Ramscar et al., 2014). While noting that there are changes in performance on many psychometric tasks with aging, the results of this long series of studies indicate that these changes reflect the “consequences of learning on information processing, and not cognitive decline.” Across the years adults develop a greater sensitivity to small details of differences in stimuli, accumulate more acquired knowledge, and, as a result, have more and different demands in their memory search “which escalate as experience grows.” These researchers concluded that the performance of older adults is a reflection of predictable outcomes of increased learning on information processing and is not an indication of cognitive decline. Viewing cognitive differences across the lifespan as related to better awareness of details and accumulated learning is an example of a hopeful perspective which could effectively empower any individual to factor neuroplasticity into their efforts to promote improved business and health behaviors.

Researchers have referred to Michael Merzenich as the “father of plasticity” because he enjoyed a long career that established that the human brain is highly plastic and that led Merzenich to develop science-based novel interventions to drive improvements. Because of her decades of research preceding that of Merzenich, let us consider Marian Diamond to be the “mother of neuroplasticity.” Her work influenced a paradigm shift for scientists when she was the first to prove that the brain shrinks with impoverishment and grows in an enriched environment at any age (Diamond et al., 1971, 1984; Malkasian and Diamond, 1971). Fred Gage brought science another paradigm shift with the proof of neurogenesis in humans (Eriksson et al., 1998); showing how animals could sustain fivefold induction of neurogenesis (Kempermann et al., 2002); demonstration of how humans can increase neurogenesis (Pereira et al., 2007); and using human induced pluripotent stem cells to model neurogenesis in the hippocampus (Yu et al., 2014). Richard Davidson’s research found that thought alone was associated with neuroplastic gains (Davidson and Lutz, 2008; Davidson and McEwen, 2012) and improved immune response (Davidson et al., 2003; Kaliman et al., 2014).

There is a growing corpus of literature on ways to drive brain plasticity in a positive direction that could contribute more powerfully in strategies of intervention for healing and enhancement of function than would research on what drives loss. These findings coupled with recent neuroscience clearly showing the potential for improving brain plasticity (Goh and Park, 2009) could give humans unprecedented hope for personal empowerment. Neuroplasticity research has fleshed out what these chemical, anatomical, and performance gains could include.

Healthcare providers including psychologists (Cramer et al., 2011) need to address all of the essentials identified by Diamond as well as other lifestyle choices such as sleep (Jacobs et al., 2004; Guzman-Marin and McGinty, 2006; Zhu et al., 2012; Castronovo et al., 2014; Irwin, 2014), reducing inflammation (Kohman and Rhodes, 2012; Rosano et al., 2012; Irwin, 2014) and turning stress into power (Sapolsky, 2004; Zucconi et al., 2006). Psychology is uniquely positioned to maximize the “revolution which is set to transform the diagnosis and treatment of mental illness and reverse the lack of major progress made in curbing associated ill health and death over the past 100 years” (White, 2011). This specialty can influence “the development of a credible risk score coupled with some, or all of, cognitive training, psychosocial approaches, education, and the use of specially designed video and computer games” as well as promoting choices that can enhance neuroplasticity for, per Marian Diamond, “Enriching Heredity.”

Applying interventions in positive ways to enhance neuroplasticity is the model proposed herein because it captures changes made in the Diamond lab; the only change in their enriched environment was having lab workers hold and talk with the rats; that “TLC” resulted in fifty percent increase in longevity while maintaining plastic gains even in animals that lived to *the equivalent of ninety human years* (Diamond et al., 1984). Diamond opined these plastic gains could be enjoyed by humans at any age. She identified five essentials for a healthy brain: newness, challenge, exercise, diet, and love.

## Newness and Challenge

Animal and human research have shown that environmental stimulation is critical for enhancing and maintaining cognitive function. Novelty, focused attention and challenge are essential components of enhancing cognitive function (Mahncke et al., 2006a,b; Houillon et al., 2013). Perceived challenge is associated with enjoyment of the task (Abuhamden and Csikszentmihalyi, 2012); it functions as reinforcement for humans.

Even when additional stimulation was not provided until rats were middle-aged, an enriched environment resulted in a fivefold increase in neuronal phenotypes that was associated with “significant improvements in learning parameters, exploratory behavior, and locomotor activity” (Kempermann et al., 2002). In addition, these new and enriching experiences resulted in decreased age dependent degeneration as shown by less accumulation of lipofuscin in the dentate gyrus. These findings corroborate those of Diamond’s work that neuroplastic gains with new and enriched environments are not limited to a brief “critical” period (Malkasian and Diamond, 1971; Uylings et al., 1978).

The timing of enrichment has also been the focus of studies with humans such as the Mayo Clinic Study of Aging (Vemuri et al., 2014) showing that academic and career achievements in the 75th percentile showed higher levels of cognition with a delay of cognitive impairment of 8.7 years as compared to individuals in the 25th percentile. This research involved 1,995 people without dementia aged 70–89, of whom only 277 had mild cognitive impairment (MCI). Since people whose intellectual enrichment was focused in midlife also showed significant gains, newness and challenge can be seen as a powerful way to lengthen the “healthy lifespan.”

In the Rush Memory and Aging Project the 964 individuals without cognitive impairment had an average age of 78.7 years and an average of 14.6 years of education when they agreed to annual evaluation and brain autopsy at their death (Wilson et al., 2014). In the beginning of the study they were asked if by the age of 18 they had foreign language instruction; if they had any music lessons; and if so, how many years of each. Over the course of about 6 years of annual examinations, 396 individuals developed MCI. The risk of MCI was about 30% less in those who before the age of 18 had had more than 4 years of foreign language training; the same finding was true of those who prior to 18 years of age had had more than 4 years of music lessons. When compared to people who had neither foreign language training nor music lessons before turning 18, the risk of MCI was about 60% less in individuals who had more than 4 years of both foreign language instruction and music lessons in their early years. It is unknown how much of this could have been mediated by the extent to which individuals used these skills during the intervening years between youth and being in the study.

Music is a complex and multisensory form of enrichment that has a positive influence on neuroplasticity in several regions of the brain because it requires integration of audiovisual information as well as appreciation of abstract rules (Paraskevopoulos et al., 2012; Kuchenbuch et al., 2014). Magnetoencephalography measures with individuals with an average age of 26.45 found that the anterior prefrontal cortex played a central role and that the neuroplastic response was greater in musicians with long term training than was noted in those with short term training (Paraskevopoulos et al., 2014). After 4 months of piano lessons, people aged 60–84 years enjoyed improved mood as well as significant improvements in the cognitive skills of attention, control, motor function, visual scanning, and executive functioning (Seinfeld et al., 2013). A recent review (Benz et al., 2016) found music training associated with enhanced cognition in a variety of musical and non-musical skills “spanning from executive functions to creativity.”

Non-musician healthy individuals aged 60–84 responded to the background music of Mozart with significant increase in their processing speed in comparison to the other three groups that had silence, white noise or the background music of Mahler (Bottiroli et al., 2014). In the same study, both Mozart and Mahler as background music was associated with better memory as compared to the groups tested with silence or white noise in the background. These scientists opined that the emotions induced by the music facilitated the memory advantage. Young, non-musician university students that listened to music while encoding had better word recognition than peers who were kept in silence (Ferreri et al., 2013). Enhanced activation of the dorsolateral prefrontal cortex correlated with enhanced encoding and retrieval in those listening to music. Community-dwelling humans 65 years old and older were tested initially and again in 6 months. Those who participated weekly in an hour-long music-based exercise class during the 6 months had decreased anxiety and gained in the executive skill of resisting interference as compared to the control group (Hars et al., 2014).

Japanese individuals 65 and older enjoyed physical exercise with musical accompaniment for 1 h once a week completing 40 h in 1 year (Satoh et al., 2014). With each session the exercise intensity was gradually increased. Half of the group heard the music played in harmony with the exercise. The other half only heard percussion that kept the beat while the people read the lyrics without music while exercising. While both groups may have appreciated gains in psychomotor speed, only the music group had significant improvement in visuospatial function. These scientists believe that cognitive functioning in elders can be enhanced when music is combined with physical exercise.

To what extent is neurogenesis relevant to information processing? New brain cells in the dentate gyrus are essential for discriminating fine differences in experiences and sensory inputs (Jessberger and Gage, 2014). It is this awareness of differences in small details that picks up newness. It is during their sixth to eighth week that new neurons are more excitable than mature brain cells (Ge et al., 2007) permitting the plastic response of stimulated adult-born new brain cells while preserving existing neuronal function. This important period is when the per cent of new brain cells that survive and get integrated (Tashiro et al., 2007) can be influenced by intrinsic and extrinsic factors (Dranovsky et al., 2011). It is also when less strong exciting currents are essential to cause a plastic response such as survival, integration, memory, or long term potentiation. These new brain cells are also thought to hold associations between time-related experiences that may not be related in content like those that occur during memory flashbacks.

In their first few days of life new brain cells grow dendrites that reach into the dentate gyrus and axons are evident. It is during this young and excitable period that new neurons require experiences such as physical activity and learning that includes challenge and newness to become integrated and stable in the dentate gyrus. Although the many steps between birth and the connectivity that includes synaptic integration are still awaiting definition, there is largely no difference between mature brain cells and newborn ones by the time new neurons are about 8 weeks old (Deshpande et al., 2013). While many of the steps and mechanisms of such rapid maturity are still being studied (Jessberger and Gage, 2014), “current hypotheses suggest that it will depend on both intrinsic signaling pathways and extrinsic regulators and local network activity.”

Working with adults aged 60–89 using a computer training program, the duration of and presentation speed of stimuli adapted to the skill level of the person in order to maintain a degree of challenge (Berry et al., 2010). As a result of practice and challenge, individuals had improved working memory for tasks they had not been trained on. Pre-training and post-training electroencephalography also showed functional brain plasticity. The sample size was small (*N* = 32) and well educated (13–21 years of education). Further research is indicated on how to maximize transfer of gains from training programs that are computer based, provide adaptive challenge and can reinforce progress with greater accuracy and effectiveness than could ever be managed by human trainers.

Ball et al. (2002) found that as little as ten 60- to 75-min sessions over a 5- to 6-week period of cognitive training helped normal elderly humans improve function on the specific skills of training with effect sizes that were “comparable with or greater than the amount of longitudinal decline that has been reported in previous studies;” this suggests that “these interventions have the potential to reverse age-related decline.” These humans received training in reasoning, memory, or speed of processing. Computerized speed of processing training showed immediate gains regardless of age, gender, mental status, health status or education with gains maintained over 5 years (Ball et al., 2013). Participants having four 1 h booster sessions at 11 and 35 months had gains beyond those found at immediate measures and “counteracted nearly 5 months of normative age-related decline in processing speed.” With completion of all training and booster sessions, these people demonstrated about 2.5 standard deviations of gain in their speed of processing. Ten years after their initial training, gains were evident in the targeted cognitive skills of reasoning and speed of processing but not for memory. All participants at 10 years reported less difficulty with instrumental activities of daily living (Rebok et al., 2014). This was a randomized, controlled, single blind trial involving 2,802 humans in six cities in the USA, the largest trial to date. A recent small study with people 65 and older who were cognitively intact found that less daily computer use was associated with a smaller percent of hippocampal volume (Silbert et al., 2016). Pairing the use of these training programs with aerobic exercise could increase brain gains (Anderson-Hanley et al., 2012).

## Exercise

It is clear that physical exercise prescriptions need to be part of healthcare to enhance brain health (Hallal and Lee, 2013; Ryan and Nolan, 2016). Without such, the harms that can accrue are similar to those of smoking and obesity. Willey et al. (2016) found that, independent of vascular risk factors, low or no leisure physical activity was associated with greater decline in processing speed and episodic memory across 5 years as compared to individuals with moderate to heavy intensity of physical exercise.

Regular exercise can reinvigorate the immune system (Simpson et al., 2012). In elders without known cognitive impairment exercise can improve cognitive performance (Angevaren et al., 2008). Being aerobic up to 60 min can improve information processing. A recent review suggests that different types of physical activity and a high level of physical fitness can decrease the so called “normal age-related” atrophy of the hippocampus and increase volume of the hippocampus (Niemann et al., 2014). With elders whose average age was 83, it predicted greater integrity in microstructures in brain networks related to memory (Tian et al., 2014). It can result in greater health of brain white matter in people aged 60–78 (Burzynska et al., 2014). Exercise energizes motor responses to improve the speed of reaction (Audiffren et al., 2008). Aerobic exercise has been associated with increased neurogenesis in humans (Pereira et al., 2007). Exercise influences survival and maturation of adult born neurons (Snyder et al., 2009). In community dwellers between the ages of 55 and 80, physical exercise was a powerful method to increase gray matter volume in the hippocampus and prefrontal cortex “effectively reversing age-related loss in volume by 1–2 years” with related improvements in memory performance (Erickson et al., 2011). Six months of high intensity aerobic exercise with women between the ages of 55 and 85 who had mild cognitive decline was a potent non-pharmacologic treatment that improved their performance on multiple tests of executive functioning (Baker et al., 2010). A review of random controlled trials suggested that physical exercise could be a powerful way to increase gray brain matter in elders such that cognitive losses and behavioral problems associated with brain atrophy can be prevented (Erickson et al., 2014). It may have pervasive benefits that could translate into less risk in humans for Alzheimer’s disease (Voss et al., 2013).

All things considered it is URGENT to prescribe exercise at any age (Colcombe and Kramer, 2003) because it can be considered beneficial for body and brain (Diamond et al., 1971; Malkasian and Diamond, 1971; Eriksson et al., 1998; Gage, 2002; Pereira et al., 2007; Angevaren et al., 2008; Larson, 2008; Snyder et al., 2009; Lojovich, 2010; Voss et al., 2010, 2013; Erickson et al., 2011, 2014; Kohman and Rhodes, 2012; Simpson et al., 2012; Hallal and Lee, 2013; Barnard et al., 2014; Burzynska et al., 2014; Jessberger and Gage, 2014; Nagamatsu et al., 2014; Tian et al., 2014; Gajewski and Falkenstein, 2016; Ryan and Nolan, 2016). There is no reason to hold back on these prescriptions while evolving research attempts to establish guidelines on preferred dose, timing and method.

In the Cardiovascular Health Study, calorie expenditure was measured along with assessments of cognitive functioning and MRI measurements of brain volume (Raji et al., 2016) in individuals with an average age of 78.3. Their findings suggest “that simply caloric expenditure, regardless of type or duration of exercise, may alone moderate neurodegeneration and even increase GM volume in structures of the brain central to cognitive functioning.”

## Diet and Inflammation

Total intake of food and fluid, frequency of intake and content consumed all factor into the molecular events of energy metabolism and neuroplasticity (Gomez-Pinilla and Tyagi, 2013). The optimal combination of nutrients can be a practical way of enhancing cognitive performance while increasing the health span. Given the scope of this review, full coverage of all pertinent research is not possible in this writing. The intent of this writing is to highlight several dietary choices that could be neuroprotective (Dauncey, 2014), could have positive effects on neuroplasticity (Murphy et al., 2014) including on adult neurogenesis (Chesnokova et al., 2016), and could influence the reduction of chronic inflammation (Barbaresko et al., 2013) which has deleterious effects on brain health and function.

Calorie restriction with adequate nutrients has been associated with health benefits through increased longevity in organisms from yeast to flies, worms, and mammals. Research suggests that *hara hachi bu*, or “eat until you are 80% full,” has been an important factor in exceptional longevity with increased health span for one human population (Willcox et al., 2014). Reducing calories 30% was associated with an average of 20% improvement in verbal memory after 3 months (Witte et al., 2009). Some of these cognitive and general health benefits of calorie restriction in humans are thought to be related to the reduction of inflammation and oxidative damage. The brain and body interaction in response to the restriction of calories could influence the sleep cycle which has been associated with inflammation; and it might be related to “the global metabolic reprogramming of the central biological clock” (Fusco and Pani, 2013). Still, the molecular nuances of the calorie restriction remains poorly understood. The problem with low grade inflammation that can be chronic as well as undiagnosed is that it can be associated with decreased cognitive functioning with aging (Marioni et al., 2009; Calcada et al., 2014).

Intermittent fasting in animals found benefits that equaled or exceeded the benefits of calorie restriction; brain cells in these animals were more capable of resisting the injury of an injection into the hippocampus that has known toxic effects (Anson et al., 2003). Reducing caloric intake seems to improve synaptic resilience to damage and modify the number, architecture, and performance of synapses (Rothman and Mattson, 2013). A reduction in inflammation with better preservation of cognitive function in animals with sepsis suggested that intermittent fasting can induce adaptive responses systemically as well as in the brain (Vasconcelos et al., 2014).

Neither calorie restriction nor intermittent fasting should be taken lightly. Each require healthy nutrition. Whether that uses the Okinawan Diet, that was one of the factors in the lives of high-functioning centenarians (Willcox et al., 2014), or the Mediterranean Diet, which has some evidence for being “a potential strategy to reduce cognitive decline in older age” (Knight et al., 2016), is discretionary. Since both emphasize vegetables, fruits, fish as a source of protein, and low glycemic load, both would be rich in polyphenols and the healthier polyunsaturated fats and would have antioxidant and anti-inflammatory benefits.

Interestingly, the polyphenol resveratrol also increases longevity (Dauncey, 2014; Witte et al., 2014) while preserving memory and hippocampal microstructure. This polyphenol occurs naturally in grapes, purple grape juice and some berries such as blueberries and cranberries.

Other invaluable polyphenols that get much wider dietary acceptance are the flavonoids found in cocoa which are noted for powerful anti-inflammatory as well as antioxidant effects. The added benefits of these flavonoids are the dose dependent improved blood flow to the brain as well as increased health and flexibility of blood vessels (Monahan et al., 2012). A recent review (Nehlig, 2013) of the neuroprotective effects of the flavonoids in cocoa suggested that they “provoke angiogenesis, neurogenesis and changes in neuron morphology, mainly in regions involved in learning and memory.” Another review (Latif, 2013) similarly found that cocoa flavonoids are neuroprotective and can enhance mood and cognitive function. Humans aged 50–69 years who consumed 900 mg of cocoa flavanols daily for 3 months enjoyed improved dentate gyrus performance on cognitive testing as well as on fMRI (Brickman et al., 2014).

Curcumin is a neuroprotective polyphenol with antiinflammatory and antioxidant capacity that can increase differentiation of neural stem cells into neurons in rats (Chen et al., 2014). It has shown a capacity to enhance neurogenesis and increase the number of neural stem cells in the hippocampus of adult mice (Kim et al., 2008). Healthy humans aged 60–85 appreciated improvements in cognition and mood (Cox et al., 2015).

Also crucial to optimal central nervous system structure and function are the essential omega-3 fatty acids eicosapentaenoic acid (EPA) and docosahexaenoic acid (DHA; Barberger-Gateau, 2014) which humans cannot create. Primary sources include fish and plant foods. Interestingly, in the parts of the brain essential to cognition and memory one study found increased gray matter volumes that were associated with fish consumption and independent of plasma measures of omega-3 fatty acids (Raji et al., 2014), highlighting the complexity of, and interactions of, dietary impact in humans. An animal study (Tyagi et al., 2015) found that a diet early in life that was high in omega-3 fatty acids protected brain cells from environmental challenges later in life. When these animals were transitioned from high omega-3s to a Western diet, the “epigenetic memory” protected these animals from cognitive decline. In the Framingham Heart Study people whose DHA level was in the top quartile had a highly significant 47% lower risk for developing dementia (Schaefer et al., 2006).

A random controlled trial (Boespflug et al., 2016) that supplemented with fish oil found “increased red blood cell omega-3 content, working memory performance, and BOLD signal in the posterior cingulate cortex during greater working memory load in older adults with subjective memory impairment.” Thus they suggested that supplementing with omega-3 fish oil could enhance brain cell response to challenges in working memory.

A research review suggests that a high brain concentration of DHA can optimize synaptic plasticity and efficiency and help maintain homeostasis in the synapses (Denis et al., 2013). For efficient transmission of data between brain cells, the plasma membrane must remain fluid. DHA is a component of this membrane. Rats maintained on a mixture of alpha-linolenic and linoleic acid showed enhanced learning that the researchers opined was due to the fatty acids changing the amount of cholesterol in the membrane of the neurons (Yehuda et al., 1998). Adequate intake of the essential fatty acids is crucial to maintaining the fluid transmission of molecules across neuronal membranes because this is where much of the action takes place for such core brain functions as learning, memory, and sleep (Yehuda et al., 2002). They are also essential in building the myelin sheath that enhances efficient processing of information (Yehuda et al., 2005).

The finding that DHA is vulnerable to oxidative damage underscores both the need for polyphenols as well as the complexity of the interactive neuroplastic influence of the several components of the dietary intervention matrix which also needs to consider essential vitamins and minerals. For example, since brain health requires adequate Vitamin B_12_, episodic measures of this status are recommended (Barnard et al., 2014).

## Love, Perception, and Reduced Stress

Perhaps love is one of the most valuable intentional emotional experiences humans can produce to drive brain plasticity in a positive direction. It is significant that the only change in experimental paradigm in the Diamond research lab was the addition of “TLC” in holding and talking with rats (Diamond et al., 1984) and that this TLC resulted in continued production of neuroplastic gains through a 50% increase in lifespan to the human equivalent of 90 years.

Diamond believed that the same gains could be appreciated in older humans but a confounding variable to consider is human perception. In the Baltimore Longitudinal Study of Aging stereotypes were assessed in people without dementia decades prior to when annual magnetic imaging and brain autopsies were done (Levy et al., 2016). Their findings were rather motivating (or sobering, depending on your perspective): “Those holding more-negative age stereotypes earlier in life had significantly steeper hippocampal volume loss and significantly greater accumulation of neurofibrillary tangles and amyloid plaques, adjusting for relevant covariates.” The cognitive skills of verbal fluency and memory (Robertson et al., 2016) were also found to decline over 2 years associated with “negative perceptions of aging.” The impact of stress that would be associated with negative perceptions was not addressed in these studies.

Richard Davidson brought affective neuroscience to the forefront by showing the neuroplastic gains in humans associated with thought (Davidson and Lutz, 2008; Ferrarelli et al., 2013). Mindfulness meditation could have antiinflammatory influences similar to those targeted by prescription drugs with a faster response noted in experienced meditators (Kaliman et al., 2014).

A study of long-term meditators and controls between the ages of 24 and 77 years, suggested that practitioners of long term meditation may have less atrophy of brain gray matter with aging (Luders et al., 2015). Their average was 20 years of meditation with a range from 4 to 46 years. While there may be many confounding variables associated with a successful long term meditation practice, the study does add “support to the hypothesis that meditation is brain-protective and associated with reduced age-related tissue decline” and that could benefit brain structure throughout the brain. White brain matter plasticity has even been found with short periods of meditation (Tang et al., 2012). A review of social influences on neuroplasticity (Davidson and McEwen, 2012) described interventions that can reduce stress and promote wellbeing as well as prosocial behaviors. These include studies of meditation on compassion, mindfulness and kindness with associated functional and structural changes.

A rich social network of friends and family stimulates and enhances healthy aging. In contrast, neurogenesis in animals is reduced with stress and depression. In humans hippocampal volume loss is predicted by depression but not age (Sheline et al., 1999). Training such as cognitive therapy and meditation could enhance wellbeing and other prosocial elements of the human experience and positive plasticity (Davidson and McEwen, 2012).

## Sleep

Mice showed decreased function in the blood brain barrier with sleep deprivation (He et al., 2014). This interface between circulation and the brain is crucial to adequate supply of nutrients and oxygen to brain cells. Sleep deprivation in mice resulted in neuroinflammation in the hippocampus and associated deficits in learning and memory (Zhu et al., 2012). A primary role of sleep may be to restore brain energy metabolism since wakefulness consumes more energy particularly in gray brain matter (Plante et al., 2014).

A study of 6,050 adults aged 65 or older (Spira et al., 2014) found greater quality of life and independent functioning in individuals who had adequate sleep as compared to those who reported insomnia. Chronic insomnia in humans was associated with hippocampal atrophy that suggests decreased neurogenesis; this was associated with cognitive deficits (Joo et al., 2014). In a population of 2,822 men aged 67 and older, measured and reported sleep disturbance was associated with cognitive decline.

A review (Guzman-Marin and McGinty, 2006) found that accumulated sleep deprivation and sleep fragmentation greater than 24 h was associated with a decrease in neurogenesis that was not quickly reversible. Seventeen people that had never complied with treatment of chronic obstructive sleep apnea showed “diffuse reduction” in white brain matter integrity that was associated with cognitive dysfunction as measured with neuropsychological testing (Castronovo et al., 2014). However, after 1 year of compliance with treatment, brain pathology has improved significantly along with “significant improvements involving memory, attention, and executive-functioning.” Memory deficits and mood effects noted with sleep deprived humans may have some association with impaired neurogenesis (Mueller et al., 2013).

Chronic sleep deprivation in animals resulted in increased inflammatory molecules and decreased BDNF which is crucial to many components of neuroplasticity (Zielinski et al., 2014). A recent review of animal research (da Costa Souza and Ribeiro, 2015) concludes that the major physiological challenge created by sleep deprivation can include “cognitive deficits, inflammation, general impairment of protein translation, metabolic imbalance, and thermal deregulation.” Sleep is essential for removal of waste and distribution of “glucose, lipids, amino acids, growth factors, and neuromodulators” (Jessen et al., 2015). One of the neuroprotective mechanisms of adequate sleep may be its reduction of inflammation that can be associated with aging (Irwin, 2014) as well as with decreased neurogenesis as observed in animal models (Guzman-Marin and McGinty, 2006). However, when one night of sleep deprivation in rats included gentle handling to prevent sleep, neurogenesis increased significantly initially as well as 15 and 30 days later (Zucconi et al., 2006).

## Discussion

We are gifted to be alive in the age of technology; to have decades of research on neuroplasticity; to have an increasing database on evidence-based interventions associated with improving health; and to read findings suggesting that “age-related cognitive decline may be slowed, arrested or even reversed.” Some of the most convincing and compelling research comes from animal studies showing the capacity of positive interventions to drive neuroplasticity in a positive direction. Diamond et al. (1984) had already found significant gains in brain architecture and performance in rats when she responded to the challenge that they were not elderly by adding an impactful intervention; technicians removed rats from their cage, held them and talked to them. The result of this “TLC” was 50% increase in lifespan to the equivalent of 90 human years. Perhaps most important, rats continued to show brain gains across this longer lifespan; Diamond opined that humans could appreciate these same brain gains at any age. Equally convincing on the value of the power of positives is the work by Zucconi et al. (2006). Rather than finding the usual detrimental effects of sleep deprivation, gentle handling of rats to prevent sleep was associated with significant increases in neurogenesis initially, 15 days later and 30 days later. Caution is essential in generalizing these findings to humans. Based on animal research it was assumed that neurogenesis declines rapidly after a certain age (Jessberger and Gage, 2008). Humans show less decline in neurogenesis with aging and still produce 700 new brain cells in each hippocampus each day (Spalding et al., 2013), according to innovative research using Carbon-14 dating. The rate of neurogenesis in animals and in humans has been increased by factors including aerobic exercise and sustained fivefold with long-term environmental enrichment (Kempermann et al., 2002). Human research might consider the impact of some form of “TLC” such as using one hand to massage the other hand during those over-night flights, night shift work or other circumstances that include sleep deprivation.

An example of neuroplastic flexibility in humans is the “significant improvements involving memory, attention, and executive-functioning” after 1 year of compliance with CPAP treatment in people whose previous non-compliance with treatment of sleep apnea showed “diffuse reduction” in white brain matter integrity that was associated with cognitive dysfunction (Castronovo et al., 2014). Even though this was a small sample, it might help clinicians influence motivation and empower clients to increase healthy sleep for brain health. Essential molecules for health must circulate in adequate supply to the brain and waste products that could have toxic impact must be removed for best brain performance and maintenance. That is part of the critical function of sleep as shown in research on the glymphatic system (Jessen et al., 2015).

Building on animal research on neuroplastic gains associated with exercise, the finding of “effectively reversing age-related loss in volume by 1–2 years” in the hippocampus and prefrontal cortex with associated improvements in memory (Erickson et al., 2011) might also increase motivation. Coupled with multiple other studies showing improvements in brain chemistry, architecture, and performance this body of research is an invitation to apply concepts and techniques in clinical practice to educate as well as increase treatment compliance with this and other non-pharmacological interventions that can be powerful, portable, and inexpensive ways of enhancing brain chemistry and architecture while improving general health. Human research has the added advantage of considering the impact of thought alone. Whether long term meditation for tens of thousands of hours (Davidson and Lutz, 2008) or short term for 4 weeks (Tang et al., 2012), structural and functional neuroplasticity have been observed.

Although calorie restriction and some nutrients have been associated with increased healthy longevity in many species, this could require greater creativity in motivating individuals to eat less and eat differently. Educating people that some cognitive and general health benefits could be related to the resultant reduction in inflammation and oxidative damage may not be enough. More research on the Okinawa Program should be encouraged because the World Health Organization declared Okinawa a centenarian center of the world based on the percentage of Okinawans that remained physically, socially and cognitively intact well past the age of 100, a distinction that persisted until Western lifestyle choices markedly changed that demographic. With the growing global burden of obesity and associated negative health impacts, clinicians could play pivotal roles in empowering people to celebrate benefits of *hara hachi bu* for their brain health; to structure reinforcement schedules for adherence to healthier food choices; and to learn from the Okinawan model that led to remarkable vigorous longevity.

Extensive work by Karlene Ball and Michael Merzenich has used behavioral techniques in computer software training that could adapt to the current level of functioning such that positive reinforcement was predominant. Merzenich has repeatedly shown that brain plasticity can be influenced in positive ways that can enrich human experience (Merzenich, 2013) suggesting that “age-related cognitive decline may be slowed, arrested, or even reversed” (Mahncke et al., 2006a). Both Ball and Merzenich have found significant gains in skills that were trained. It is especially significant that these enduring positive effects of neuroplasticity based computerized cognitive training have been found to impact such important issues as quality of life (Wolinsky et al., 2014), driving mobility (Edwards et al., 2009), independence (Jobe et al., 2001), improved memory and attention in humans aged 65 and older (Smith et al., 2009; Zelinski et al., 2011), and reduced risk of increased depression 1 and 5 years after baseline training (Wolinsky et al., 2009). Thus, ample research exists to indicate the need for behavioral techniques in neuroplasticity based computerized programs in healthcare. The finding of Ball and associates that some gains persisted up to 10 years highlights the need for research on dose, timing, and how broadly these gains might influence other factors such as memory, judgment, and prosocial behaviors.

Since computerized complex new learning coupled with aerobic exercise has shown increased gains over either intervention alone in early research (Anderson-Hanley et al., 2012), ballroom dancing could be another research avenue for measuring cognitive gains in an activity that combines at least physical and cognitive elements; add social aspects when dancing with others. A recent review (Gajewski and Falkenstein, 2016) suggests that combining cognitive training with aerobic exercise in natural activities like dancing might be the most beneficial because it is multilevel with both physical and cognitive coordination required.

Since aerobic exercise is associated with increased neurogenesis in humans, integration of these new brain cells requires newness and challenge, research has shown increased survival rates of new brain cells in animals afforded an enriched environment, and type of stimulation during aerobic exercise can influence the site of integration as well as improved cognition related to those efforts, the timing is right for increased research to develop computerized interventions that can be used in association with aerobic activity, whether on the dance floor or cardio machine, to assess potential academic, cognitive, prosocial, and business benefits across a healthier longevity.

Some humans increase their probability of retaining brain architecture and performance while thriving on hope and purpose whereas others maintain “more negative age stereotypes earlier in life” with the unfortunate result of “significantly steeper hippocampal-volume loss and significantly greater accumulation of neurofibrillary tangles and amyloid plaques, adjusting for relevant covariates” with associated decline over 2 years in verbal fluency and memory (Robertson et al., 2016). In contrast a small study that embedded purposeful activity within a program in which elders volunteered to support the academic success of children “forestalled and possibly reversed age-related declines in annual rates of atrophy” in the “cortical and hippocampal volumes” of the participants compared to controls (Carlson et al., 2015). This is another example of an intervention that encourages further research on purpose, service, and potentially confounding variables such as the increased physical and social activity.

Age alone does not explain individual differences in cognitive decline, volume in specific brain areas, nor the association between these variables and quality of life and survival. Many factors have been associated with driving neuroplasticity in a positive direction. By empowering clients to use these evidence based interventions, we have the potential to increase health span as well. Much of the research referenced herein can be seen as capturing the perspective of positive psychology which “studies what makes life most worth living” and “is a call for psychological science and practice to be as concerned with strength as with weakness; as interested in building the best things in life as in repairing the worst; and as concerned with making the lives of normal people fulfilling as with healing pathology (Peterson, 2008).”

What has become clear is that human brains remain plastic, i.e., responsive to intrinsic and extrinsic stimulation, throughout our lifespan. Very complex interactions exist between physiology, function, social, and psychological factors.

Baker et al. (2012) noted that, although high intensity physical exercise resulted in normal adults being “less vulnerable to the pathological effects of an unhealthy diet,” individuals with MCI appreciated even greater benefit from a “healthy diet on Aβ modulation” when they also included high intensity physical exercise in their efforts. These researchers concluded that exercise “may thus interact with diet to alter pathological processes that ultimately modify AD risk.” That is particularly empowering in light of the later study (Baker, 2015) in which individuals aged 70 or older who maintained 70–80% of their maximum heart range for 45 min a day, 4 days a week for 6 months appreciated reduced phosphorylated tau levels in their cerebrospinal fluid with associated cognitive skill gains compared to a group whose exercise protocol was to stretch.

The complexity of all of these interactions needs to be acknowledged, even emphasized. This is eloquently stated by Aimone et al. (2014): “In short, while the technological advances of the last couple of decades have revealed most of what we know about adult neurogenesis, we may yet learn that we still know very little.”

The timing is urgent for humans worldwide to apply new neuroscience even while many of these promising research advances are in their infancy. With increasing lifespan has come the increase in the number of people suffering cognitive decline. Within the so-called “normal age-related cognitive decline” there is reason to separate the myth (Ramscar et al., 2014) from the serious losses. The goals of our time can be to add dignity to the human condition *at any age*; to emphasize science based interventions with potential for enriching heredity, reversing losses and empowering individuals to drive neuroplasticity in a positive direction; to maximize human potential in ways previously unimagined; and to enjoy influencing human intelligence to evolve in a positive direction with the goal of creating more Outliers (Gladwell, 2011) who celebrate cerebral successes throughout vigorous longevity. If these efforts succeed in delaying the onset of dementia by as little as 1 year (Brookmeyer et al., 2007), the worldwide burden of Alzheimer’s disease could be reduced by as much as 9,200,000 by 2050.

Perhaps the ideal research now would be multifaceted. It would begin with informing individuals in our global community about the potential gains in brain chemistry, architecture and performance suggested in the current neuroscience database. Include the fact that even the gold standard random controlled trial is only a probability statement. Devise an easy manner of assessing in a community the current level of that community’s functioning, knowledge about this research, unique values and current use of any of the elements of these findings toward their specific goals. Encourage healthcare oversight for modifications that reflect their unique situation. Train interested individuals in how to use positive reinforcement of successive approximation of the several interventions that their community endorses. Develop measures to assess baseline and progress in the community as well as in the trainers given the finding of the Baltimore study that purpose-driven service can also include neuroplastic benefits in trainers (Carlson et al., 2015).

Current estimates may be significantly below actual occurrence of dementia since they tend to only capture the data of clinicians aware of the need to screen for dementia in the fraction of the population that accessed care. However, it is conceivable that an effective program of teaching and training trainers in the use of positive behavioral techniques and perspectives to increase knowledge and compliance with the several evidence-based interventions thus far identified could improve the global statistics on cognition.

The message of hope and empowerment is well stated by Jessberger and Gage (2008): “Despite the dramatic reductions in hippocampus-dependent function that accompany advancing age, there is also striking evidence that even the aged brain retains a high level of plasticity. Thus, one promising avenue to reach the goal of successful aging might be to boost and recruit this plasticity, which is the interplay between neural structure, function, and experience, to prevent age-related cognitive decline and age-associated comorbidities.”

The timing is urgent for clinicians to use positive behavioral techniques, measure, motivate, strategize, and increase compliance with new neuroscience associated with driving neuroplasticity in a positive direction.

## Author Contributions

The author confirms being the sole contributor of this work and approved it for publication.

## Conflict of Interest Statement

The author declares that the research was conducted in the absence of any commercial or financial relationships that could be construed as a potential conflict of interest.
